# New Genotype of Dengue Type 3 Virus Circulating in Brazil and Colombia Showed a Close Relationship to Old Asian Viruses

**DOI:** 10.1371/journal.pone.0007299

**Published:** 2009-10-13

**Authors:** Victor Hugo Aquino, Alberto Anastacio Amarilla, Helda Liz Alfonso, Weber Cheli Batista, Luiz Tadeu Moraes Figueiredo

**Affiliations:** 1 Departamento de Análises Clínicas, Toxicológicas e Bromatológicas, Faculdade de Ciências Farmacêuticas, Universidade de São Paulo, Ribeirão Preto, São Paulo, Brasil; 2 Centro de Pesquisa em Virologia, Faculdade de Medicina de Ribeirão Preto, Universidade de São Paulo, Ribeirão Preto, São Paulo, Brasil; 3 Centro de Pesquisa em Medicina Tropical, Porto Velho, Rondônia, Brasil; Institute of Infectious Disease and Molecular Medicine, South Africa

## Abstract

Dengue type 3 genotype V viruses have been recently detected in Brazil and Colombia. In this study, we described another Brazilian isolate belonging to this genotype. Phylogenetic analysis including dengue type 3 viruses isolated worldwide showed that Brazilian and Colombian viruses were closely related to viruses isolated in Asia more than two decades ago. The characteristic evolutionary pattern of dengue type 3 virus cannot explain the close similarity of new circulating viruses with old viruses. Further studies are needed to confirm the origin of the new dengue type III genotype circulating in Brazil and Colombia.

## Introduction

Dengue virus type 3 was re-introduced into the Americas (Nicaragua and Panama) in 1994 and subsequently spread into most of the Latin American and the Caribbean countries [1]. DENV-3 is classified into five genetically distinct groups [2], [3]. The DENV-3 that is circulating in the Americas belongs to the genotype III and was probably originated from an ancestor in Sri Lanka [4]. DENV-3 was introduced into Rio de Janeiro, Brazil, in December of 2000, and in January of 2002 was responsible for a severe epidemic outbreak [5]. In a recent molecular epidemiology study, we have found that DENV-3 genotype III currently circulates in several regions of Brazil and Paraguay [6]. We have also isolated a genotype V virus (D3BR_PV7_03 strain) from a fatal case of DHF in Porto Velho, Amazon Region of Brazil, in 2003, suggesting that viruses of this genotype circulates in Brazil (Aquino VH and others, unpublished data). This fact was recently confirmed, firstly, by Barcelos and colleagues in Belo Horizonte, Southeast Region of Brazil, and latterly, by Nogueira and colleagues, in Porto Velho [5], [7]. In addition, viruses of the same genotype have bee also detected in Colombia [8].

The present study analyses the phylogenetic relationship of genotype V viruses isolated in Brazil and Colombia with dengue type 3 viruses isolated worldwide.

## Materials and Methods

### Virus and E, NS1 and 3′UTR sequencing

D3BR_PV7_03 strain, which was isolated in C6/36 cells from the serum sample of a DHF patient with a fatal outcome in Porto Velho, had the envelope (E) and nonstructural 1 (NS1) genes, and the untranslated 3′ region (3′UTR) sequenced as previously reported [6]. All procedures were performed in order to avoid any kind of contamination; different rooms were used for virus isolation, RNA purification, protein E and NS1 genes amplification, and PCR product analysis. The sequences were deposited in the GenBank (E: EU570161; NS1: FJ481174; 3′UTR: FJ481175).

### Sequences analysis

The protein E gene sequence of D3BR_PV7_03 was aligned with those obtained by Barcelos and colleagues (BH_24_2003, BH_19_2003, BH_16_2003, and MG_20_2004) and by Nogueira and colleagues (BRDEN3_RO1_02 and BR DEN3_RO2_02) [7], [9]. Barcelos and colleagues have sequenced 1023 nucleotides of the protein E gene of four viruses isolated in Belo Horizonte. Therefore, to maintain the same sequence size, we decided to carry out the alignment with 1023 nucleotides of the DENV-3 E gene. The alignment included worldwide DENV-3 E gene sequences retrieved from the GenBank. Alignment was carried out with the CLUSTAL W program and the best fit-model of nucleotide substitution was selected under the hierarchical likelihood ratio test (hLTR) using the Modeltest v3.7 [10]. The phylogenetic relationships among strains were reconstructed by the neighbor-joining (NJ), maximum parsimony (MP) maximum likelihood and Bayesians methods using PAUP 4.0B10 program and MrBayes 3.1.2 [11], [12]. Distance matrixes were generated using the Tamura Nei model for nucleotide and PAM model for amino acid using the MEGA 4.0 program [13].

We have also used 306 nucleotides of E/NS1 junction to include Colombian strains as well as NS1 and UTR3′ regions to analyze the phylogenetic relationship of the viruses.

#### Ethical statement

Serum sample was collected before death and sent to our laboratory for routine dengue diagnosis. This study was approved by the Ethical Committee of the Pharmaceutical Sciences Faculty of Ribeirao Preto (Proc. 46/2005).

## Results and Discussion

**Figure 1:**
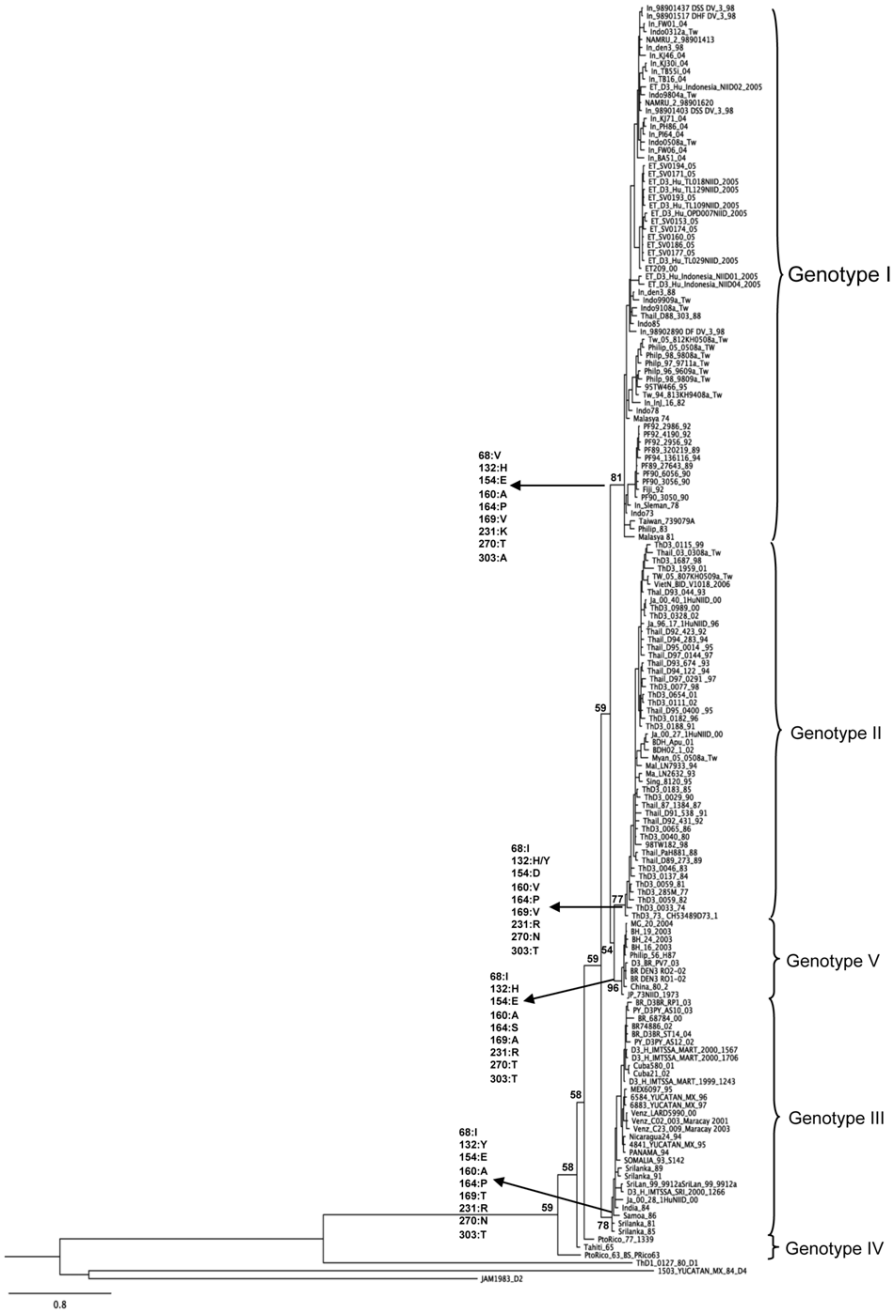
Bayesian phylogenetic trees derived from 154 global samples of DENV-3 E gene partial sequences (1023 nucleotides) inferred with MrBayes program. The posterior probabilities are expressed in percent and indicated at important nodes. DENV-1 (ThD1_0127_80_D4, AY732411), DENV-2 (JAM1983_D2, AY484605) and DENV-4 (1503_YUCATAN_MX_84_D4, DQ341212) were used as outgroup. Horizontal branch lengths are drawn to scale. Aligned sequences were analyzed in the MrModeltest 2.3 program to identify the best fit-model of nucleotide substitution for Bayesian phylogenetic reconstruction [23]. The nucleotide substitution model used was under a General Time Reversible model of nucleotide substitution with gamma-distributed rate variation (G = 1.2411) and a proportion of invariable sites (I = 0.3656) (GTR+G+I), using Akaike's Information Criterion (AIC) [24]. Five runs of 4 chains each (one cold and tree heated, temperature = 0.20) were run for 1.5×10^6^ generations, with a burn-in of 6000 generations. Characteristic amino acid substitutions at important nodes are indicated. GenBank accession numbers: BH 16 2003 (EF625832), BH 19 2003 (EF625833), BH 24 2003 (EF625834), BR 00 68784 (AY038605), D3BR RP1 03 (DQ118877), BR74886 02 (AY679147), China 80 2 (AF317645), Cuba21 02 (AY702031), D3BR PV7 03 (EU570161), BR DEN3 RO1 02 (EF629373), BR DEN3 RO2-02 (EF629373), ET ET209 00 (EF440434), ET_D3_Hu_TL129NIID_2005 (AB214882), ET_D3_Hu_TL018NIID_2005 (AB214879), ET_D3_OPD007NIID 2005 (AB219131), ET SV0153 05 (DQ453981), ET SV0160 05 (DQ453980), ET SV0174 05 (DQ453973), ET SV0177 05 (DQ453972), ET SV0193 05 (DQ453970), ET SV0194 05 (DQ453969), ET_D3_TL029NIID_2005 (AB214880), Fiji 92 (L11422), PF89 27643 89 (AY744677), PF89 320219 89 (AY744678), PF90 3050 90 (AY744679), PF90 3056 90 (AY744680), PF90 6056 90 (AY744681), PF92 2956 92 (AY744682), PF92 2986 92 (AY744683), PF94 136116 94 (AY744685), India 84 (L11424), Indo0312a TW 03 (DQ518677), Indo0508a_TW (DQ518678), IN 9108a_Tw 91 (DQ518674), IN 9909a_Tw 99 (DQ518675), IN BA51 04 (AY858037), IN DEN3 98 (AY858039), IN FW01 04 (AY858040), IN FW06 04 (AY858041), IN KJ30i 04 (AY858042), Indo_KJ71 04 (AY858044), ET_D3_Indonésia_NIID01 2005 (AB219137), ET_D3_Indonésia_NIID02 2005 (AB219138), ET_D3_Indonésia_NIID04 2005 (AB219139), IN PH86 04 (AY858045), IN PI64 04 (AY858046), IN Sleman 78 (AY648961), IN TB16 04 (AY858047), IN TB55i 04 (AY858048), Indo_73 (L11425), Indo_78 (L11426), Indo_85 (L11428), JP 73NIID 1973 (AB111085), Malasya 74 (L11429), Malasya 81 (L11427), D3_H_IMTSSA_MART_2000_1567 (AY099338), D3_H_IMTSSA_MART_2000_1706 (AY099339), D3_H_IMTSSA_MART_1999_1243 (AY099337), MG 20 2004 (EF625835), MEX6097_95 (AY146763), 6584_YUCATAN_MX 96 (DQ341203), Nicaragua 94 (AY702033), Panama 94 (DQ341209), Philip 56 H87 (L11423), Philp_05_0508aTw (DQ518673), Philp_96_9609aTw (DQ518668), Philp_98_9808aTw (DQ518671), Philp_98_9809aTw (DQ518669), PtoRico 63 (L11433), D3PY AS10 03 (DQ118883), SAMOA_86 (L11435), SOMALIA 93 S142 (DQ341208), SriLan 99 9912a (DQ518679), D3_H_IMTTSSA_Sri_2000_1266 (AY099336), SriLanka 81 (L11431), SriLanka 85 (L11436), SriLanka 89 (L11437), SriLanka 91 (L11438), Venez_C02 003_Maracay_2001 (DQ367720), Venez C23 009 Maracay_2001 (DQ367721), Venz_LARD5990_00 (AY146764), In_98901437 DSS DV_3_98 (AB189126), In_98901517 DHF DV_3_98 (AB189127), In_98901403 DSS DV_3_98 (AB189125), NAMRU_2 98901620 (AY265857), Tw_05_812KH0508a_Tw (DQ518672), ET_SV0171_05 (DQ453974), In_den3_88 (AY858038), Thail_D88_303_88 (AY145714), In_98902890 DF DV_3_98 (AB189128), 95TW466_95(DQ675519), Tw_94_813KH9408a_Tw (DQ518667), In_InJ_I6_82 (DQ401694), PF92_4190_92 (AY744684), Taiwan_739079A (AY776329), BR_D3BR_ST14_04 (DQ118882), PY_D3PY_AS12_02 (DQ118884), Cuba580_01 (AY702030), 6883_YUCATAN_MX_97 (DQ341204), 4841_YUCATAN_MX_95 (DQ341202), Ja_00_28_1HuNIID_00 (AB111081), PtoRico_77_1339 (AY146761), Tahiti_65 (L11439), ThD3_1687_98 (AY676348), ThD3_0115_99 (AY676387), ThD3_1959_01 (AY676402), ThD3_0328_02 (AY676383), ThD3_0989_00 (AY676414), ThD3_0077_98 (AY676389), ThD3_0654_01 (AY676394), ThD3_0111_02 (AY676420), ThD3_0188_91 (AY676367), ThD3_0182_96 (AY676369), Thail_03_0308a_Tw (DQ518660), TW_05_807KH0509a_Tw (DQ518659), VietN_BID V1018_2006 (EU482462), Thal_D93_044_93 (AY145720), Ja_00_40_1HuNIID_00 (AB111082), Ja_96_17_1HuNIID_96(AB111084), BDH_Apu_01 (AY656672), Thail_D92_423_92 (AY145718), Thail_D94_283_94 (AY145723), Thail_D95_0014 _95(AY145724), Thail_D97_0144_97 (AY145729), Thail_D93_674 _93 (AY145721), Thail_D94_122 _94(AY145722), Thail_D97_0291 _97(AY145730), Thail_D95_0400 _95 (AY145725), Ja_00_27_1HuNIID_00 (AB111080), BDH02_1_02 (AY496871), Myan_05_0508a_Tw, DQ518666), Mal_LN7933_94 (AY338494), Ma_LN2632_93 (AF147459), Sing_8120_95 (AY766104), ThD3_0029_90 (AY676421), ThD3_0183_85 (AY676368), Thail_87_1384_87 (AF533079), Thail_D91_538_91 (AY145717), Thail_D92_431_92 (AY145719), ThD3_0065_86 (AY676354), ThD3_0040_80 (AY676359), 98TW182_98 (DQ675520), Thail_PaH881_88 (AF349753), ThD3_0046_83 (AY676358), Thail_D89_273_89 (AY145715), ThD3_0137_84 (AY676371), ThD3_0059_81 (AY676356), ThD3_285M_77 (AY676384), ThD3_0059_82 (AY676355), ThD3_0033_74 (AY676360), 1503_YUCATAN_MX_84_D4 (DQ341212), JAM1983_D2 (AY484605), ThD1_0127_80 _D1 (AY732411).

**Figure 2:**
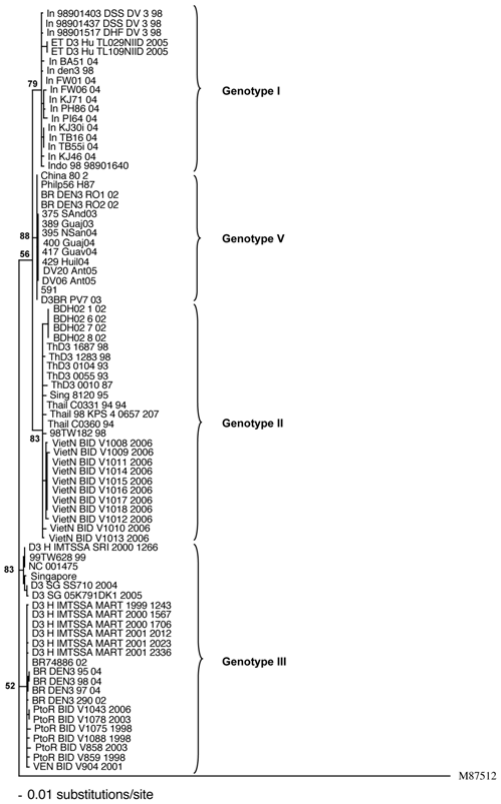
Maximum Likelihood phylogenetic tree derived from 80 global samples of DENV-3 using 306 nucleotides of E/NS1 junction with a bootstrap analysis of 500 replicates. A DENV-1 strain (M87512) was used as outgroup. Horizontal branch lengths are drawn to scale. Aligned sequences were analyzed in the Modeltest 2.3 program and found that the best fit-model of nucleotide substitution for phylogenetic reconstruction was Tamura & Nei (TrN+I) with a proportion of invariable sites (I) of 0.5203 and gamma distribution with equal rates for all sites, using Akaike's Information Criterion (AIC). GenBank accession numbers: In_98901403_DSS_DV_3_98 (AB189125), In_98901437_DSS_DV_3_98 (AB189126), In_98901517_DHF_DV_3_98 (AB189127), In_FW01_04 (AY858040), In_FW06_04 (AY858041), In_KJ30i_04 (AY858042), In_KJ71_04 (AY858044), In_PH86_04 (AY858045), In_PI64_04 (AY858046), In_TB16_04 (AY858047), In_TB55i_04 (AY858048), In_BA51_04 (AY858037), In_den3_98 (AY858039), ET_D3_Hu_TL109NIID_2005 (AB214881), China_80_2_ (AF317645), BR_DEN3_RO1_02 (EF629370), BR_DEN3_RO2_02_ (EF629373), BDH02_1_02 (AY496871), BDH02_7_02 (AY496877), ThD3_0104_93_ (AY676350), ThD3_0055_93_ (AY676351), Thail_C0331_94_94 (AY876494), ThD3_0010_87_ (AY676352), VietN_BID_V1008_2006 (EU482452), VietN_BID_V1009_2006 (EU482453), VietN_BID_V1011_2006 (EU482455), VietN_BID_V1014_2006 (EU482458), VietN_BID_V1015_2006 (EU482459), VietN_BID_V1016_2006 (EU482460), VietN_BID_V1017_2006 (EU482461), VietN_BID_V1018_2006 (EU482462), VietN_BID_V1010_2006 (EU482454), VietN_BID_V1012_2006 (EU482456), VietN_BID_V1013_2006 (EU482457), Sing_8120_95 (AY766104), D3_H_IMTSSA_SRI_2000_1266 (AY099336), NC_001475 (NC_001475), Singapore (AY662691), D3_SG_SS710_2004 (EU081181), D3_SG_05K791DK1_2005 (EU081182), BR74886_02 (AY679147), BR_DEN3_95_04 (EF629366), BR_DEN3_97_04 (EF629367), BR_DEN3_98_04_ (EF629368), BR_DEN3_290_02 (EF629369), PtoR_BID_V1043_2006 (EU482555), PtoR_BID_V1078_2003 (EU482564), PtoR_BID_V1075_1998 (EU482563), PtoR_BID_V1088_1998 (EU482566), PtoR_BID_V859_1998 (EU482596), VEN_BID_V904_2001 (EU482612), PtoR_BID_V858_2003 (EU482595), D3/Hu/TL029NIID/2005 (AB214880), Indo_98_98901640 (AY912455), In KJ46 (AY858045), Philp56 H87 (L11423), 375 And03 (EU003494), 389 Guaj03 (EU003495), 395 NSan04 (EU003496), 400 Guaj04 (EU003497), 417 Guav04 (EU003498), 429 Huil04 (EU003499), 591 DV20 Ant05 (EU003513), DV06 Ant05 (EU003514), C0360 94 (AY923865), ThD3 1283 98 (AY676349), 98TW182 (DQ675520), Thail 98 KPS 4 0657 207 (AY912458), 99TW628 99 (DQ675533), D3 H IMTSSA MART 1999 1243 (AY099337), D3 H IMTSSA MART 2000 1567 (AY099338), D3 H IMTSSA MART 2000 1706 (AY099339), D3 H IMTSSA MART 2001 2012 (AY099340), D3 H IMTSSA MART 2001 2336 (AY099342), D3 H IMTSSA MART 2001 2023 (AY099341), BDH02_8_02 (AY496878), BDH02_6_02 (AY496876), ThD3_1687_98 (AY676348), DENV1 (M87512).

The phylogenetic relationship using all approaches yielded trees with identical or nearly identical topologies; Figure 1 shows the tree constructed by the Bayesian method. The phylogenetic tree showed the characteristic distribution of dengue viruses into five genotypes [2], [3]. Viruses isolated in Porto Velho and Belo Horizonte are closely related to each other and to viruses isolated in Asia (Philippine-56, China802 and JP73NIID1973 strains) within the genotype V (Figure 1). Colombian strains have only 306 nucleotides of E/NS1 junction sequenced [8]; therefore, we used the same genomic region to analyze the phylogenetic relationship of the DENV-3. Figure 2, constructed by the Maximum Likelihood method, shows that Colombian strains belong also to genotype V, and are closely related to Brazilian strains.

**Figure 3:**
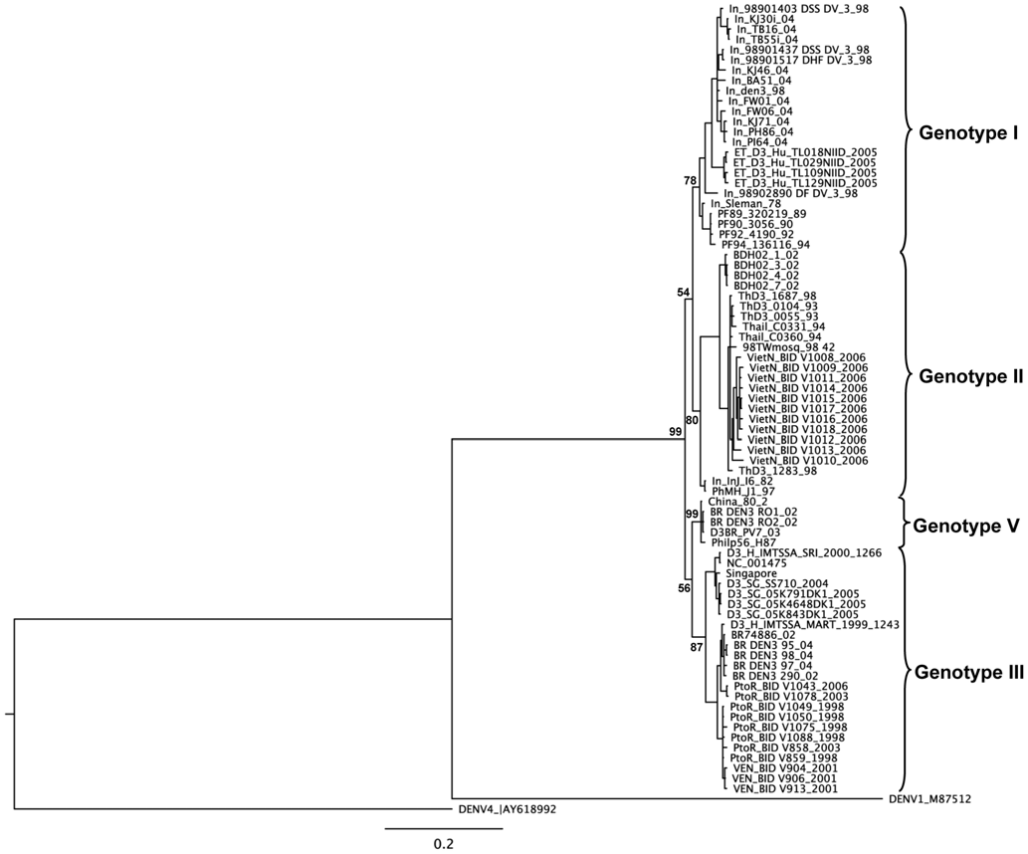
Bayesian phylogenetic trees derived from 79 global samples of DENV-3 NS1 gene sequences inferred with MrBayes program. The posterior probabilities expressed in percent are indicated at important nodes. DENV-1 (M87512) and DENV-4 (AY618992) strains were used as outgroup. Horizontal branch lengths are drawn to scale. Aligned sequences were analyzed in the MrModeltest 2.3 program to identify the best fit-model of nucleotide substitution for Bayesian phylogenetic reconstruction. The nucleotide substitution model used was under a General Time Reversible model of nucleotide substitution with gamma-distributed rate variation (G = 1.9241) and a proportion of invariable sites (I = 0.4401) (GTR+G+I), using Akaike's Information Criterion (AIC). Five runs of 4 chains each (one cold and tree heated, temperature = 0.20) were run for 1.5×10^6^ generations, with a burn-in of 6000 generations. GenBank accession numbers: D3BR PV7 03 (FJ481174), In_98901403_DSS_DV_3_98 (AB189125), In_98901437_DSS_DV_3_98 (AB189126), In_98901517_DHF_DV_3_98 (AB189127), In_98902890_DF_DV_3_98 (AB189128), ET_D3_Hu_TL018NIID_2005 (AB214879), ET_D3_Hu_TL109NIID_2005 (AB214881), ET_D3_Hu_TL029NIID_2005 (AB214880), ET_D3_Hu_TL129NIID_2005 (AB214882), China_80_2_ (AF317645), D3_H_IMTSSA_SRI_2000_1266 (AY099336), D3_H_IMTSSA_MART_1999_1243 (AY099337), BDH02_1_02 (AY496871), BDH02_3_02 (AY496873), BDH02_4_2 (AY496874), BDH02_7_02 (AY496877), In_Sleman_78 (AY648961), Singapore (AY662691), ThD3_0104_93_ (AY676350), ThD3_0055_93_ (AY676351), BR74886_02 (AY679147), PF89_320219_89 (AY744678), PF90_3056_90 (AY744680), PF92_4190_92 (AY744684), PF94_136116_94 (AY744685), In_BA51_04 (AY858037), In_den3_98 (AY858039), In_FW01_04 (AY858040), In_FW06_04 (AY858041), In_KJ30i_04 (AY858042), In_KJ71_04 (AY858044), In_PH86_04 (AY858045), In_PI64_04 (AY858046), In_TB16_04 (AY858047), In_TB55i_04 (AY858048), Thail_C0331_94_94 (AY876494), In_InJ_16_82 (DQ401690), PhMH_J1_97 (DQ401695), BR_DEN3_95_04 (EF629366), BR_DEN3_97_04 (EF629367), BR_DEN3_98_04_ (EF629368), BR_DEN3_290_02 (EF629369), BR_DEN3_RO1_02 (EF629370), BR_DEN3_RO2_02_ (EF629373), D3_SG_SS710_2004 (EU081181), D3_SG_05K791DK1_2005 (EU081182), D3_SG_05K843DK1_2005 (EU081187), D3_SG_05K4648DK1_2005 (EU081225), VietN_BID_V1008_2006 (EU482452), VietN_BID_V1009_2006 (EU482453), VietN_BID_V1010_2006 (EU482454), VietN_BID_V1011_2006 (EU482455), VietN_BID_V1012_2006 (EU482456), VietN_BID_V1013_2006 (EU482457), VietN_BID_V1014_2006 (EU482458), VietN_BID_V1015_2006 (EU482459), VietN_BID_V1016_2006 (EU482460), VietN_BID_V1017_2006 (EU482461), VietN_BID_V1018_2006 (EU482462), PtoR_BID_V1043_2006 (EU482555), PtoR_BID_V1049_1998 (EU482558), PtoR_BID_V1050_1998 (EU482559), PtoR_BID_V1075_1998 (EU482563), PtoR_BID_V1078_2003 (EU482564), PtoR_BID_V1088_1998 (EU482566), PtoR_BID_V858_2003 (EU482595), PtoR_BID_V859_1998 (EU482596), VEN_BID_V904_2001 (EU482612), VEN_BID_V906_2001 (EU482613), VEN_BID_V913_2001 (EU482614), Philip56_H87 (M93130), NC_001475 (NC_001475), ThD3_1687_98 (AY676348), 98TWmosq_98 (DQ675532), ThD3_1283_98 (AY676349), In_KJ46_04 (AY858043), Thail C0360 94 (AY923865), DENV1 (M87512), DENV4 (AY618992).

**Figure 4:**
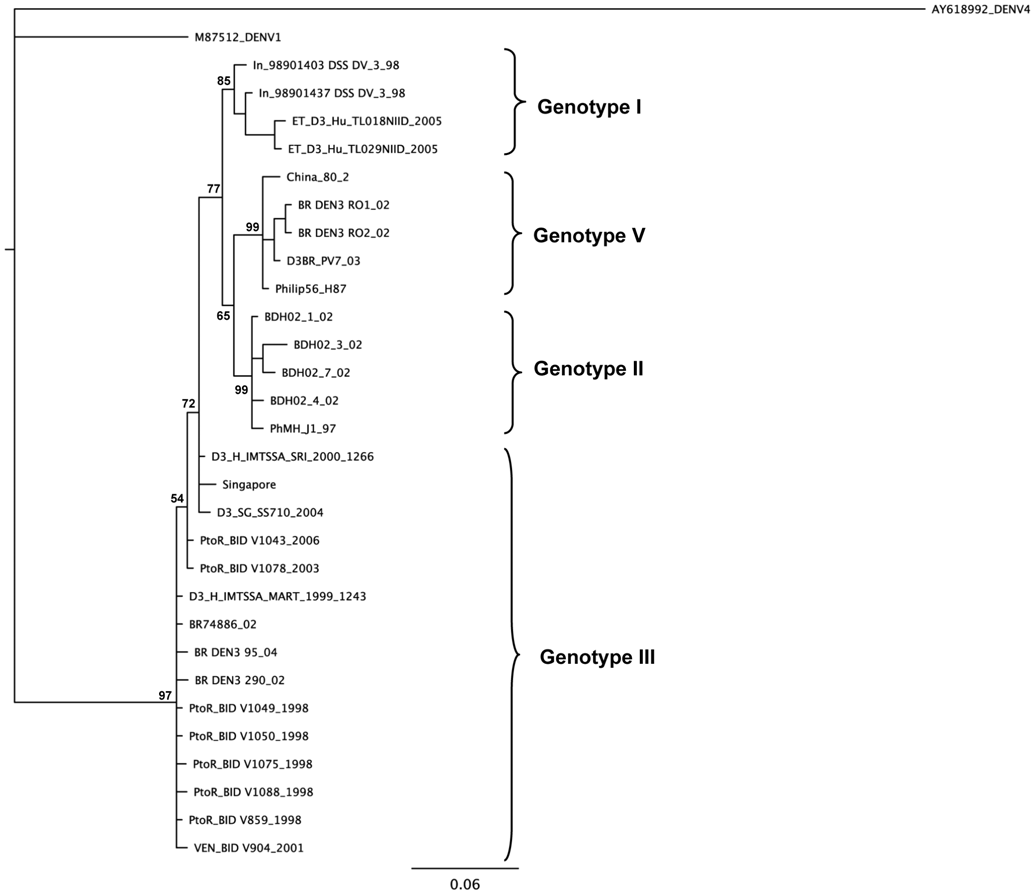
Bayesian phylogenetic trees derived from 31 global samples of DENV-3 based on the 3′UTR sequences inferred with MrBayes program. The posterior probabilities expressed in percent are indicated key nodes. The DENV-1 (M87512) and DENV4 (AY618992) strains were used as outgroup. Horizontal branch lengths are drawn to scale. Aligned sequences were analyzed in the MrModeltest 2.3 program to identify the best fit-model of nucleotide substitution for Bayesian phylogenetic reconstruction. The nucleotide substitution model used was under a General Time Reversible model of nucleotide substitution with a proportion of invariable sites (I = 0.6264) (GTR+I), using Akaike's Information Criterion (AIC). Five runs of 4 chains each (one cold and tree heated, temperature = 0.20) were run for 1.5×10^6^ generations, with a burn-in of 6000 generations. GenBank accession numbers: D3BR PV7 03 (FJ481175), In_98901437_DSS_DV_3_98 (AB189126), In_98901403_DSS_DV_3_98 (AB189125), ET_D3_Hu_TL018NIID_2005 (AB214879), ET_D3_Hu_TL029NIID_2005 (AB214880), China_80_2_(AF317645), D3_H_IMTSSA_MART_1999_1243 (AY099337), D3_H_IMTSSA_SRI_2000_1266 (AY099336), BR74886_02 (AY679147), Philip56_H87 (M93130), BR_DEN3_95_04 (EF629366), BR_DEN3_290_02 (EF629369), BR_DEN3_RO1_02 (EF629370), BR_DEN3_RO2_02_(EF629373), D3_SG_SS710_2004 (EU081181), Singapore (AY662691), PtoR_BID_V859_1998 (EU482596), PtoR_BID_V1088_1998 (EU482566), PtoR_BID_V1078_2003 (EU482564), PtoR_BID_V1075_1998 (EU482563), PtoR_BID_V1043_2006 (EU482555), PtoR_BID_V1049_1998 (EU482558), PtoR_BID_V1050_1998 (EU482559), VEN_BID_V904_2001 (EU482612), PhMH_J1_97 (DQ401695), BDH02_1_02 (AY496871), BDH02_3_02 (AY496873), BDH02_4_2 (AY496874), BDH02_7_02 (AY496877), DENV1 (M87512), DENV4 (AY618992).

To further analyze the phylogenetic relationship of the new genotype circulating in Brazil, NS1 and 3′UTR genomic regions were also analyzed. Colombian strains do not have these regions sequenced. Brazilian strains showed again a close relationship with viruses within the genotype V (Figure 3 and 4).

To estimate the percent divergence of dengue type 3 viruses, a distance matrix of nucleotide and amino acid sequences, based on the 1023 nucleotides of E gene, was constructed (Table 1). The distance matrix showed a higher percent divergence for viruses from different genotypes and proportional to the isolation date, i.e., the greater the difference in age of isolates, the higher the percent divergence, except for viruses within genotype V. Brazilian strains showed to be very similar to each other, with a maximum divergence of 0.8% at the nucleotide level between the furthest sequences, BH_24_2003 and D3BR_PV7_03, which translates into a 1.8% divergence at the protein level. Brazilian strains showed also a high similarity with Asian strains within the genotype V, with a maximum divergence of 0.9% at the nucleotide level (1.8% at the amino acid level) between the furthest sequences, BH_24_2003 and JP_73NIID_1973 (Table 1). Sequence comparison of the E gene of the earliest American genotype III viruses on record, Nicaraguan and Panaman strains isolated in 1994, with the suggested ancestors, Sri Lankan strains isolated between 1981 and 1989, showed a divergence between 1.9% and 2.1% at the nucleotide level and between 1.5% and 0.9% at the amino acid level, respectively, higher than that observed comparing Brazilian and Asian strains within genotype V.

**Table 1:** Nucleotide and amino acid pairwise distances between DENV-3 strains used in this study for the envelope glycoprotein.

	% Amino acid divergence																							
	**1**	**2**	**3**	**4**	**5**	**6**	**7**	**8**	**9**	**10**	**11**	**12**	**13**	**14**	**15**	**16**	**17**	**18**	**19**	**20**	**21**	**22**	**23**	**24**
[**1**] ** #MG_20_2004 (2004, V)**		0.0	0.6	0.0	0.9	0.9	1.2	0.3	1.2	1.2	2.7	3.4	3.0	2.4	2.1	3.6	3.3	3.3	3.3	2.7	2.7	2.4	2.1	3.0
[**2**] ** #BH_19_2003 (2003, V)**	0.0		0.6	0.0	0.9	0.9	1.2	0.3	1.2	1.2	2.7	3.4	3.0	2.4	2.1	3.6	3.3	3.3	3.3	2.7	2.7	2.4	2.1	3.0
[**3**] ** #BH_24_2003 (2003, V)**	0.2	0.2		0.6	1.5	1.5	1.8	0.9	1.8	1.8	3.3	3.7	3.6	3.0	2.7	3.6	3.3	3.9	3.9	3.3	3.3	3.0	2.7	3.6
[**4**] ** #BH_16_2003 (2004, V)**	0.0	0.0	0.2		0.9	0.9	1.2	0.3	1.2	1.2	2.7	3.4	3.0	2.4	2.1	3.6	3.3	3.3	3.3	2.7	2.7	2.4	2.1	3.0
[**5**] ** #BR_DEN3_RO2-02 (2002, V)**	0.4	0.4	0.6	0.4		0.0	0.3	0.6	1.5	1.5	3.0	3.7	3.3	2.7	2.4	3.9	3.6	3.6	3.6	3.0	3.0	2.7	2.4	3.3
[**6**] ** #BR_DEN3_RO1-02 (2002, V)**	0.4	0.4	0.6	0.4	0.0		0.3	0.6	1.5	1.5	3.0	3.7	3.3	2.7	2.4	3.9	3.6	3.6	3.6	3.0	3.0	2.7	2.4	3.3
[**7**] ** #D3_BR_PV7_03 (2003, V)**	0.6	0.6	0.8	0.6	0.2	0.2		0.9	1.8	1.8	3.3	4.0	3.6	3.0	2.7	4.3	3.9	3.9	3.9	3.3	3.3	3.0	2.7	3.6
[**8**] ** #Philip_56_H87 (1956, V)**	0.1	0.1	0.3	0.1	0.3	0.3	0.5		0.9	0.9	2.4	3.1	2.7	2.1	1.8	3.3	3.0	3.0	3.0	2.4	2.4	2.1	1.8	2.7
[**9**] ** #China_80_2 (1980, V)**	0.6	0.6	0.8	0.6	0.6	0.6	0.8	0.5		1.2	2.7	3.4	3.0	2.4	2.1	3.7	3.3	3.3	3.3	2.7	2.7	2.4	2.1	3.0
[**10**] ** #JP_73NIID_1973 (1973, V)**	0.7	0.7	0.9	0.7	0.7	0.7	0.9	0.6	0.7		2.7	3.4	3.0	2.4	2.1	3.7	3.3	3.3	3.3	2.7	2.7	2.4	2.1	3.0
[**11**] ** #East_Timor_SV0174_05 (2005, I)**	6.7	6.7	6.9	6.7	6.9	6.9	7.1	6.5	6.9	6.7		1.2	1.5	0.9	0.6	2.7	3.0	3.6	3.6	3.0	3.0	2.6	2.3	3.3
[**12**] ** #Indonesia_NIID02_05 (2005, I)**	6.8	6.8	6.9	6.8	7.0	7.0	7.2	6.6	7.0	6.9	3.0		2.1	1.5	1.2	3.3	3.6	3.9	3.9	3.3	3.3	3.3	3.0	3.6
[**13**] ** #PF94_136116_94 (1994, I)**	6.5	6.5	6.8	6.5	6.7	6.7	7.0	6.4	6.8	6.4	5.5	5.5		1.2	0.9	4.2	3.9	4.2	4.2	3.6	3.6	3.3	3.0	3.9
[**14**] ** #Philp_0508_05 (2005, I)**	6.4	6.4	6.7	6.4	6.6	6.6	6.9	6.3	6.7	6.5	4.9	4.8	5.0		0.3	3.0	2.7	3.3	3.3	2.7	2.7	2.4	2.1	3.0
[**15**] ** #Philip_83 (1983, I)**	4.8	4.8	5.0	4.8	5.0	5.0	5.2	4.7	5.0	4.9	4.5	4.6	3.7	3.8		3.3	3.0	3.3	3.3	2.7	2.7	2.4	2.1	3.0
[**16**] ** #BDH_Jacob_01 (2001, II)**	6.6	6.6	6.6	6.6	6.9	6.9	7.1	6.5	6.9	6.5	9.5	9.1	8.9	8.7	8.2		0.3	3.0	3.0	2.4	2.4	3.3	3.0	2.7
[**17**] ** #Bang0108a_Tw (2005, II)**	6.5	6.5	6.5	6.5	6.7	6.7	6.9	6.3	6.7	6.3	9.7	9.3	8.8	8.6	8.0	0.1		3.0	3.0	2.4	2.4	3.3	3.0	2.7
[**18**] ** #Nicaragua_94 (1994, III)**	6.8	6.8	7.0	6.8	7.0	7.0	7.2	6.6	7.0	6.6	8.5	8.9	8.9	7.8	7.7	8.9	9.1		0.6	0.6	0.6	1.5	1.2	0.9
[**19**] ** #PANAMA_94 (1994, III)**	6.8	6.8	7.0	6.8	7.0	7.0	7.2	6.6	7.0	6.6	8.4	8.9	8.9	8.0	7.6	8.9	9.1	0.4		0.6	0.6	1.5	1.2	0.9
[**20**] ** #MART_00_1567 (2000, III)**	6.7	6.7	7.0	6.7	7.0	7.0	7.2	6.6	7.0	6.6	8.4	8.8	8.6	8.1	7.6	9.0	9.2	1.0	1.0		0.0	0.9	0.6	0.3
[**21**] ** #PY_03_AS10 (2003, III)**	7.0	7.0	7.3	7.0	7.2	7.2	7.5	6.9	7.3	6.9	8.2	8.9	8.3	8.0	7.4	9.3	9.5	1.2	1.2	0.4		0.9	0.6	0.3
[**22**] ** #SriLanka_81 (1981, III)**	5.5	5.5	5.8	5.5	5.7	5.7	6.0	5.4	5.8	5.4	7.4	7.8	8.1	7.0	6.4	7.7	7.9	1.9	1.9	2.1	2.4		0.3	1.2
[**23**] ** #SriLanka_85 (1985, III)**	5.6	5.6	5.8	5.6	5.8	5.8	6.0	5.4	5.8	5.4	7.2	7.5	8.2	6.8	6.0	7.8	7.9	2.0	2.0	2.3	2.5	0.6		0.9
[**24**] ** #SriLanka_89 (1989, III)**	6.7	6.7	6.9	6.7	6.9	6.9	7.1	6.5	6.9	6.5	7.8	8.7	8.7	7.6	7.2	8.4	8.6	2.1	2.1	2.4	2.4	1.4	1.5	
	% Nucleotide divergence																							

In parenthesis are indicated the year of isolation and the genotype.

The evolutionary rate (nucleotide substitutions per site per year, subs./site/yr) and the time of the most recent common ancestor (T_mrca_, years) of the viruses sampled were estimated using both strict and relaxed molecular clock models (Table 2). Evolutionary parameters were estimated by using the Bayesian Markov Chain Monte Carlo (MCMC) method implemented in BEAST v1.4.8 [14], [15]. The substitution rates of genotypes I, II and III were very similar (between 9.1×10^−4^ and 15×10^−4^ subs./site/yr) and are in agreement with previous studies [16], [17]. The substitution rate of genotype V was slightly lower (between 3.7×10^−4^ and 4.2×10^−4^ subs./site/yr) than the other genotypes and the T_mrca_ was between 1954 and 1955.

**Table 2:** Estimated substitutions rates and dates for DENV-3 genotypes.

Dataset	Molecular Clock	Substitutions site^−1^ year^−1^	*T*_mrca_, year
Genotype I	Strict	9.1×10^−4^ (7.1×10^−4^ to 11×10^−4^)	1959 (1955–1962)
	Relaxed	12×10^−4^ (9.8×10^−4^ to 14×10^−4^)	1967 (1964–1969)
Genotype II	Strict	9.6×10^−4^ (7.7×10^−4^ to 12×10^−4^)	1966 (1963–1968)
	Relaxed	13×10^−4^ (11×10^−4^ to 16×10^−4^)	1970 (1968–1972)
Genotype III	Strict	11×10^−4^ (8.2×10^−4^ to 13×10^−4^)	1976 (1974–1978)
	Relaxed	15×10^−4^ (10×10^−4^ to 20×10^−4^)	1979 (1978–1981)
Genotype V	Strict	4.2×10^−4^ (1.3×10^−4^ to 8.3×10^−4^)	1954 (1952–1956)
	Relaxed	3.7×10^−4^ (1.7×10^−4^ to 5.9×10^−4^)	1955 (1954–1956)

It is well known that dengue viruses that are circulating in the Americas have an Asian origin; however, it is intriguing how viruses that are recently circulating in Brazil and Colombia are closely related to viruses isolated in Asia more than two decades ago. It might be possible that viruses of genotype V have been maintained in nature in a low circulation rate for years being detected now. However, the low percent sequence divergence observed between the Brazilian isolates and Asian strain of genotye V is not in accordance with viruses that were circulating for such a long time. Considering that the substitution rate of genotype V was between 3.7×10^−4^ and 4.2×10^−4^ subs./site/yr (Table 2), the expected divergence [18] at the nucleotide level between the oldest isolate Philippines56 strain (isolated in 1956) and Brazilian isolates (isolated in 2004) should be between 3.5–4.0%. However, the divergence observed among these strains was 0.1%, 35 fold lower than the expected divergence. On the other hand, the first genotype III viruses detected in the Americas (Panaman and Nicaraguan strains, 1994) and the suggested ancestor (Sri Lancan strain, 1989) showed a divergence of 1.9–2.1%, which is in accordance with the expected divergence of 1.1–1.5% between these viruses (substitution rate of genotype III viruses was between 11×10^−4^ and 15×10^−4^ subtitution/site/year).

Several phylogenetic studies have been carried out to show the migratory pattern of dengue type III viruses between neighboring countries and between continents [2], [4], [6], [19], [20]. Those studies have shown that DENV-3, when introduced to a new area, evolves locally, resulting in geographically-associated clusters closely related to other virus recently circulating in other region. Interestingly, we have shown in this study that viruses recently circulating in Brazil and Colombia form a monophyletic cluster together with viruses isolated in Asia more than two decades ago. We have used partial genomic regions to analyze the phylogenetic relationship; however, Schreiber and colleagues [21], analyzing the unique Brazilian strain (BR DEN3 RO1-02, GenBank EF629370) of genotype V with the entire genome available, found the same close relationship with the Asian strains. The characteristic evolutionary pattern of DENV-3 could not explain the close relationship observed between new Brazilian and Colombian isolates with old Asian strains. One of the genotype V strains, Philippines 56 (H87), is used as a prototype of DENV-3 and it is widely distributed in several laboratories of the world. Could some how this strain escape from the laboratory and started to infect humans, thus explain the close relationship of the new viruses with genotype V strains? Other authors have previously reported evidences of human infections caused by a laboratory strain of dengue virus. Viruses isolated in several countries such as Mexico, Honduras, Cuba, China, and Vietnam were found to be more closely related to the NGC-44, a laboratory prototype of DENV-2, than to strains that were circulating in those countries [22]. However, this theory was never proved. Therefore, more studies are needed to confirm the origin of American genotype V viruses.
